# Theoretical description of halogen bonding – an insight based on the natural orbitals for chemical valence combined with the extended-transition-state method (ETS-NOCV)

**DOI:** 10.1007/s00894-012-1474-4

**Published:** 2012-06-06

**Authors:** Mariusz P. Mitoraj, Artur Michalak

**Affiliations:** Department of Theoretical Chemistry, Faculty of Chemistry, Jagiellonian University, R. Ingardena 3, 30-060 Cracow, Poland

**Keywords:** Covalency, ETS-NOCV, Halogen bonding

## Abstract

In the present study we have characterized the halogen bonding in selected molecules H_3_N–ICF_3_ (**1-NH**_**3**_), (PH_3_)_2_C–ICF_3_ (**1-CPH**_**3**_), C_3_H_7_Br–(IN_2_H_2_C_3_)_2_C_6_H_4_ (**2-Br**), H_2_–(IN_2_H_2_C_3_)_2_C_6_H_4_ (**2-H**_**2**_ **)** and Cl–(IC_6_F_5_)_2_C_7_H_10_N_2_O_5_ (**3-Cl)**, containing from one halogen bond (**1-NH**_**3**_, **1-CPH**_**3**_) up to four connections in **3-Cl** (the two Cl–HN and two Cl–I), based on recently proposed ETS-NOCV analysis. It was found based on the NOCV-deformation density components that the halogen bonding C–X^**…**^B (X-halogen atom, B-Lewis base), contains a large degree of covalent contribution (the charge transfer to X^**…**^B inter-atomic region) supported further by the electron donation from base atom B to the empty σ*(C–X) orbital. Such charge transfers can be of similar importance compared to the electrostatic stabilization. Further, the covalent part of halogen bonding is due to the presence of σ-hole at outer part of halogen atom (X). ETS-NOCV approach allowed to visualize formation of the σ-hole at iodine atom of CF_3_I molecule. It has also been demonstrated that strongly electrophilic halogen bond donor, [C_6_H_4_(C_3_H_2_N_2_I)_2_][OTf]_2_, can activate chemically inert isopropyl bromide (**2-Br**) moiety via formation of Br–I bonding and bind the hydrogen molecule (**2-H**_**2**_). Finally, ETS-NOCV analysis performed for **3-Cl** leads to the conclusion that, in terms of the orbital-interaction component, the strength of halogen (Cl–I) bond is roughly three times more important than the hydrogen bonding (Cl–HN).

**Figure:**
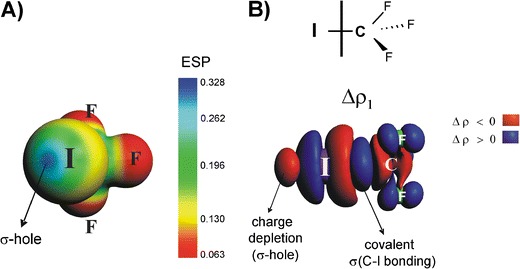
ETS-NOCV reprezentation of σ-hole at iodine together with the molecular electrostatic potential picture

ETS-NOCV reprezentation of σ-hole at iodine together with the molecular electrostatic potential picture

## Introduction

Halogen and hydrogen bonding plays an essential role in chemistry and biochemistry [1–14]. Recently, the halogen bonding, A–X^**…**^B (X–halogen atom, B–Lewis base, A–electronegative atom), attracted considerable attention due to its strong, selective and directional character [4–14]. These features make them very important in supramolecular crystal engineering and in determination of biological structures [4–13]. Therefore, many efforts are made to describe the halogen bonding by theoretical methods of quantum chemistry; this in turn can help in rational design of new halogen bonded systems with desired molecular properties [4–14].

The pioneering experimental studies reporting on the existence of halogen bonding dates back to the mid nineteenth century [15–19]. The first theoretical analysis of the resonance structures has been proposed by Mulliken to explain the molecular spectra of halogen bonded complexes [20]. One decade later Hassel and co-workers expanded this area by developing new systems containing halogen bonds [19–21]. In his Nobel lecture Hassel highlighted that the electrophilic part of halogen atom can be very crucial in molecular self-assembly phenomena. In addition, it has been stressed the importance of charge transfer in halogen bonded systems [20].

The novel concept that explains the origin of halogen bonding was proposed by Politzer and coworkers [6–9, 13]. The authors noticed for the first time, based on the molecular electrostatic potential, that there exists an electron deficiency at the outer part of the halogen atom, so called *σ-hole*, what leads in turn to the electrostatic attraction with Lewis bases. Hence, the halogen bonding is driven mainly by the electrostatic term [6–9]. Very recently Hennemann and coworkers extended the σ-hole concept to analysis of polarized hydrogen bonds [13].

In the present study we will characterize the halogen bonding in selected molecules in terms of quantitative role of the *electronic* (*the charge transfer and covalency*) and *electrostatic* factors. The recently developed ETS-NOCV scheme will be used, that originates from a combination of the extended transition state (ETS) [22] energy decomposition approach with the natural orbitals for chemical valence (NOCV) analysis [23–29]. It was shown that ETS-NOCV is able to extract and directly quantify the crucial components (σ, π, δ, etc.) that constitute various types of chemical bonds including donor-acceptor [23, 24, 26–29], covalent [25], intra-molecular agostic [30, 31] and inter-molecular hydrogen bonding [32–35].

**Fig. 1:**
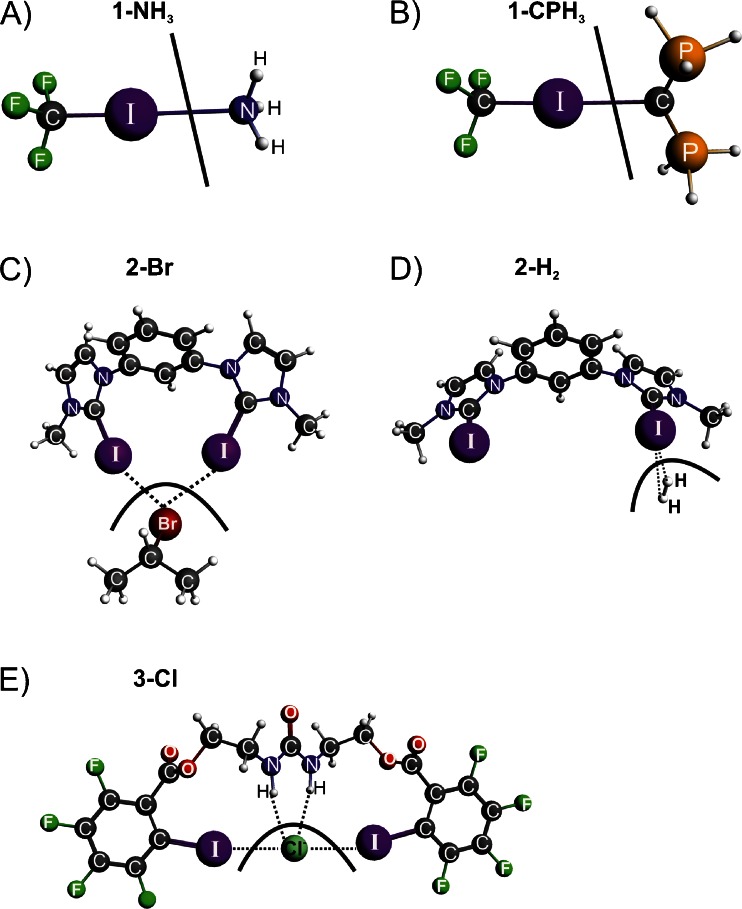
Halogen bonded systems studied in the present work. A thick black line represents the way of fragmentation used in a description of bonding

We will first apply the ETS-NOCV scheme in a description of relatively weak halogen bonds (**1-NH**_**3**_ and **1-CPH**_**3**_, see panels a and b of Fig. 1). Ammonia and divalent carbon (0) ligands [C(PH_3_)_2_] will be used as electron donating species, whereas CF_3_I molecule as electron acceptor. The CL_2_ ligands (where L is an electron donor) have recently attracted considerable attention due to untypical oxidation state of carbon atom [36–38]. ETS-NOCV approach will also be used for the first time to describe the σ-hole at iodine atom of CF_3_I, based on deformation density contributions obtained from the NOCV analysis of the bond between the CF_3_ and I fragments. In the next step, we will focus our attention on the catalytic system [C_6_H_4_(C_3_H_2_N_2_I)_2_][OTf]_2_ (**2** in Fig. 1). It has been proven experimentally that this halogen bond donor is a very strong electrophilic species that is able to break covalent C–Br bond in benzhydryl bromide (Ph_2_HC–Br) [39]. Accordingly, as the second objective of our study we will perform pioneering theoretical analysis of the interaction between isopropyl bromide (the methyl groups were used instead of phenyl rings) and the model [C_6_H_4_(C_3_H_2_N_2_I)_2_]^2+^ system (the counter anion OTf^–^ has been omitted in the calculations), see **2-Br** in Fig. 1. Further, we will demonstrate that very high electrophilicity of **2** can lead to the formation of dihydrogen complex (**2-H**_**2**_ in Fig. 1). Finally, the halogen (Cl–I) and hydrogen bonds (Cl–HN) co-existing within the same molecule (**3-Cl** in Fig. 1) will be analyzed [40]. This novel urea-based system has been recently developed experimentally by Chudzinski and coworkers [40].

## Computational details

All the DFT calculations presented here are based on the Amsterdam density functional (ADF2009) program in which ETS-NOCV scheme was implemented [41–45]. The Becke-Perdew exchange-correlation functional [46, 47] was applied with an inclusion of the dispersion correction (BP86-D) [48]. A standard triple-zeta STO basis containing two sets of polarization functions was adopted for all of the elements (TZ2P). Auxiliary *s, p, d, f,* and *g* STO functions, centered on all nuclei, were used to fit the electron density and to obtain accurate Coulomb potentials in each SCF cycle. The contours of deformation densities were plotted based on ADF-GUI interface [49].

In our analysis of halogen bonding each of the systems are divided up into two individual fragments as shown schematically by thick black lines in Fig. 1. Then we used the ETS-NOCV method to study the interaction between these subsystems. Thus, our analysis is based on the bonding between the two close shell molecular fragments.

## Computational methods

Bonding analysis presented in this study is based on the ETS-NOCV approach which is a combination of the extended transition state (ETS) [22] method with the natural orbitals for chemical valence (NOCV) scheme [23–29].

Historically, the natural orbitals for chemical valence (NOCV) have been derived from the Nalewajski-Mrozek valence theory [50–56] as eigenvectors of the deformation density matrix. It was shown [24, 26] that the natural orbitals for chemical valence pairs (*ψ*_*-k*_,*,ψ*_*k*_) decompose the differential density Δ*ρ* into NOCV-contributions (Δ*ρ*_*k*_):

1 $$ \Delta \rho (r) = \sum\limits_{{k = 1}}^{{{{M} \left/ {2} \right.}}} {{v_k}[ - \psi_{{ - k}}^2(r) + \psi_k^2} (r)] = \sum\limits_{{k = 1}}^{{{{M} \left/ {2} \right.}}} {\Delta {\rho_k}(r)}, $$

where *ν*_*k*_ and *M* stand for the NOCV eigenvalues and the number of basis functions, respectively. Visual inspection of deformation density plots (Δ*ρ*_*k*_) helps to attribute symmetry and the direction of the charge flow. Negative values of this function are marked by red color (outflow of electrons due to bond formation), whereas positive values of Δ*ρ*_*k*_ are in blue color (charge accumulation). In addition, within ETS-NOCV scheme, the deformation-density based picture is enriched by the energetic estimations, $\Delta E_{{orb}}^k$, for each Δ*ρ*_*k*_.

In the ETS energy decomposition scheme, the interaction energy Δ*E*_int_ between the fragments (exhibiting geometries as in the combined molecule) is divided into three components:

2 $$ \Delta {E_{\text{int}}} = \Delta {E_{\text{elstat}}} + \Delta {E_{\text{Pauli}}} + \Delta {E_{\text{orb}}}. $$

The first term, Δ*E*_elstat_, corresponds to the classical electrostatic interaction between the fragments as they are brought to their positions in the final molecule. The second term, Δ*E*_Pauli_, accounts for the repulsive Pauli interaction between occupied orbitals on the fragments in the combined molecule. The third stabilizing term, Δ*E*_*orb*,_ represents the interactions between the occupied molecular orbitals of one fragment with the unoccupied molecular orbitals of the other fragments as well as mixing of occupied and virtual orbitals within the same fragment (inner-fragment polarization). This energy term may be linked to the electronic bonding effect coming from the formation of a chemical bond.

In the combined ETS-NOCV scheme [30] the orbital interaction term (Δ*E*_orb_) is expressed in terms of NOCV’s eigenvalues (*v*_*k*_) as:

3 $$ \Delta {E_{{orb}}} = \sum\limits_k {\Delta E_{{orb}}^k = } \sum\limits_{{k = 1}}^{{{{M} \left/ {2} \right.}}} {{v_k}[ - F_{{ - k, - k}}^{{TS}}} + F_{{k,k}}^{{TS}}], $$

where $F_{{i,i}}^{{TS}}$ are diagonal Kohn-Sham matrix elements defined over NOCV with respect to the transition state (TS) density (at the midpoint between density of the molecule and the sum of fragment densities). The above components $\Delta E_{{orb}}^k$ provide *energetic estimation* of Δ*ρ*_*k*_, thus, they characterize the importance of a particular electron flow channel for the bonding between considered molecular fragments. As has been already mentioned, we will use the dispersion corrected BP86-D functional [46–48], hence, the dispersion correction (Δ*E*_disp_) will be added to Δ*E*_int_ values.

## Results and discussion

**Fig. 2:**
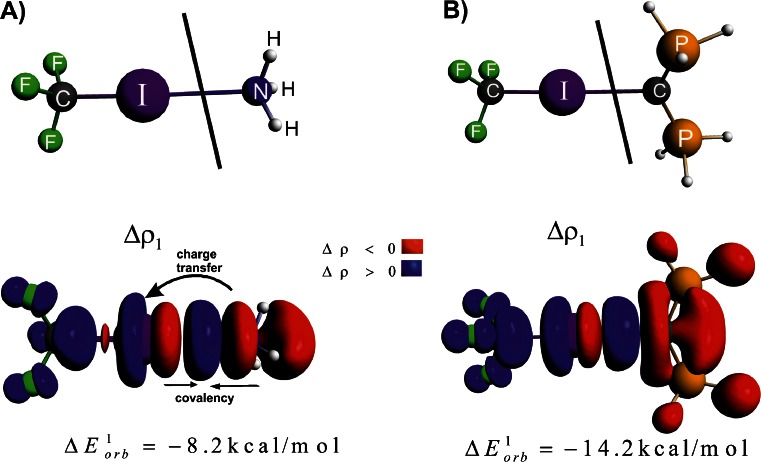
The contour of deformation density contribution Δ*ρ*_1_ describing formation of the halogen bonding in **1-NH**_**3**_ (part A) and in **1-CPH**_**3**_ (part B). In addition the corresponding ETS-NOCV-based energies (in kcal mol^-1^) are shown. The numerically smallest contour values are ±0.0005 a.u.

We will start our discussion from the NOCV’s based description of halogen bonding in **1-NH**_**3**_. It can be noticed from Fig. 2a that the leading deformation density channel, Δ*ρ*_1_, with the corresponding electronic stabilization $\Delta E_{{orb}}^1 = {{{ - 8.2kcal}} \left/ {{mol}} \right.}$, constitutes the halogen bonding between ammonia and CF_3_I molecule. Qualitatively, Δ*ρ*_1_ is based on the donation from the lone electron pair of nitrogen to the empty σ*(I-C). This transfer leads to elongation of C–I bond, by 0.015 Å, as compared to non-bonded CF_3_I species. It is important to note that of similar importance is the *covalent* contribution that originates from the electron transfer from both the nitrogen and iodine atoms to the I–N inter atomic region, see Fig. 2a. It is noteworthy that, due to formation of halogen bond, the accumulation of electron density is also observed at the carbon atom of CF_3_I, which is in line with an increase in *s*–character of carbon; this issue has been deeply analyzed recently by Grabowski using NBO method [12]. The charge transfer, Δ*ρ*_1_, characterized by $\Delta E_{{orb}}^1 = {{{ - 8.2kcal}} \left/ {{mol}} \right.}$ nearly covers the total orbital interaction term $\left( {\Delta {E_{{orb}}} = {{{ - 9.2kcal}} \left/ {{mol}} \right.}} \right)$, see Table 1. The remaining part of $\Delta E_{{orb}}^{{rest}} = {{{ - 1.0kcal}} \left/ {{mol}} \right.}$ corresponds to CF_3_I  NH_3_ back-donation (ca. 0.5 kcal mol^-1^) and the intra-fragment polarization (ca. 0.5 kcal mol^-1^).

**Table 1:** ETS-energy decomposition^a^ (in kcal mol^-1^) characterizing the halogen bonded systems (BP86-D/TZ2P)

Systems^b^	Δ*E*_Pauli_	Δ*E*_elstat_	Δ*E*_orb_	Δ*E*_disp_	Δ*E*_int_
1-NH_3_	18.3	-15.2	-9.2	-1.3	-7.4
1-CPH_3_	34.0	-27.6	-16.8	-2.8	-13.2
2-Br	22.8	-20.3	-19.6	-2.8	-19.9
2-H_2_	3.0	-1.6	-2.2	-1.0	-1.8
3-Cl	48.0	-63.8	-44.1	-2.5	-62.4

^a^ΔE_int_=ΔE_Pauli_+ΔE_elstat_+ΔE_disp_+ΔE_orb_

^b^The labeling corresponds to Fig. 1

An analysis of the data presented in Table 1 for **1-NH**_**3**_ leads to the conclusion that the electrostatic contribution (Δ*E*_*elstat*_ = -15.2 kcal mol^-1^) is visibly more important than the orbital-interaction factor (covalent + charge transfer, as discussed above, Δ*E*_orb_ = -9.2 kcal mol^-1^). Thus, our ETS-NOCV result supports the role of electrostatic factor disclosed in the previous studies based on the other theoretical approaches (electrostatic potential, symmetry adapted perturbation theory-based energy partitioning) [6–9, 57, 58]. However, it should be emphasized that both, the electrostatic and orbital-interaction components are crucial for total energetic stabilization. The total interaction energy that includes all bonding contributions, is stabilizing: Δ*E*_int_ = -7.4 kcal mol^-1^. This value is in line with other theoretical estimations available in literature [8].

**Fig. 3:**
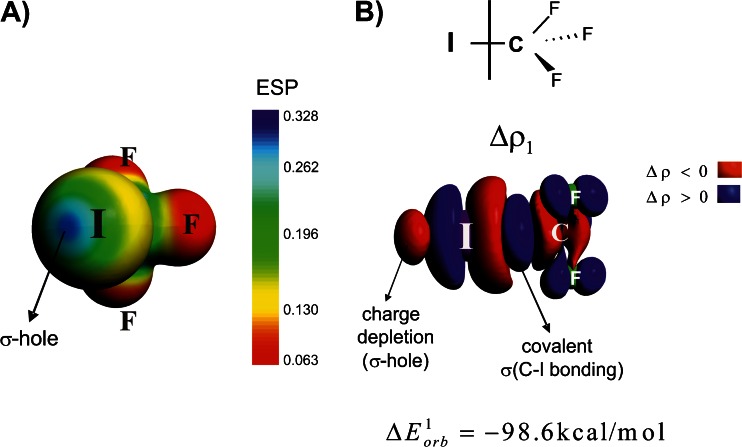
The electrostatic potential characterizing CF_3_I molecule (part A). The contour of deformation density contribution Δ*ρ*_1_ describing the formation of C–I bond in CF_3_I molecule (part B). In addition the corresponding ETS-NOCV-based energy is shown. The numerically smallest contour values are ±0.0006 a.u.

Domination of the electrostatic component can be understood in terms of σ-hole concept that has been introduced by Politzer and coworkers and extensively studied during recent years [5–9]. According to this concept halogen bonding is based on the electrostatic attraction between the Lewis base and Lewis acid parts (σ-hole) of the halogen atom [5–9]. Such electron deficiency in the outer part of iodine atom in CF_3_I (σ-hole) is manifested by positive molecular electrostatic potential, as can be seen from Fig. 3a.

An intriguing question arises at this point. Namely, whether we are able to visualize the formation of σ-hole using deformation density contributions originating from NOCV. For this purpose we have analyzed the bonding between the iodium atom and the CF_3_ radical (each carrying one unpaired electron with the opposite spin polarizations). As can be seen from Fig. 3b, the leading deformation density channel, Δ*ρ*_1_, with the corresponding energy $\Delta E_{{orb}}^1 = {{{ - 98.6kcal}} \left/ {{mol}} \right.}$, shows the formation of covalent C–I bond. In addition, it is gratifying to see an outflow of electron density from the outer area of iodine atom, which clearly corresponds to the formation of σ-hole. It should further be noted that apart from the above components, the charge accumulation at iodine is observed due to formation of C–I bond, which confirms significant charge anisotropy around this atom [4–14]. The presence of such a anisotropy is important for the reactivity, as the halogen atom can simultaneously act as electron donor and acceptor [4–14].

Let us now briefly discuss the bonding of divalent carbon (0) ligand, C(PH_3_)_2_, with CF_3_I moiety, in **1-CPH**_**3**_, see Fig. 2b. Qualitatively, the deformation density channel, Δρ_1_, exhibits similar features as in the case of **1-NH**_**3**_. Namely, formation of the covalent I–C contribution as well as the charge transfer from the lone electron pair of carbon to the empty σ*(C–I) orbital can be noted. However, these transfers correspond to significantly higher stabilization, $\Delta E_{{orb}}^1 = {{{ - 14.2kcal}} \left/ {{mol}} \right.}$, as compared to **1-NH**_**3**_ ($\Delta E_{{orb}}^1 = {{{ - 8.2kcal}} \left/ {{mol}} \right.}$). This is probably due to stronger overlap of orbitals involved in the halogen bonding interaction. Divalent carbon (0) ligands appeared to be strong electron donors, which has been recently extensively studied by Tonner and Frenking [37]. A significant overlap leading to notable electronic stabilization in **1-CPH**_**3**_ also causes the electrostatic contribution to be more pronounced when compared to **1-NH**_**3**_ (by 12.4 kcal mol^-1^), see Table 1. Accordingly, the total interaction energy, Δ*E*_int_, becomes more stabilizing, by 5.8 kcal mol^-1^ for **1-CPH**_**3**_.

**Fig. 4:**
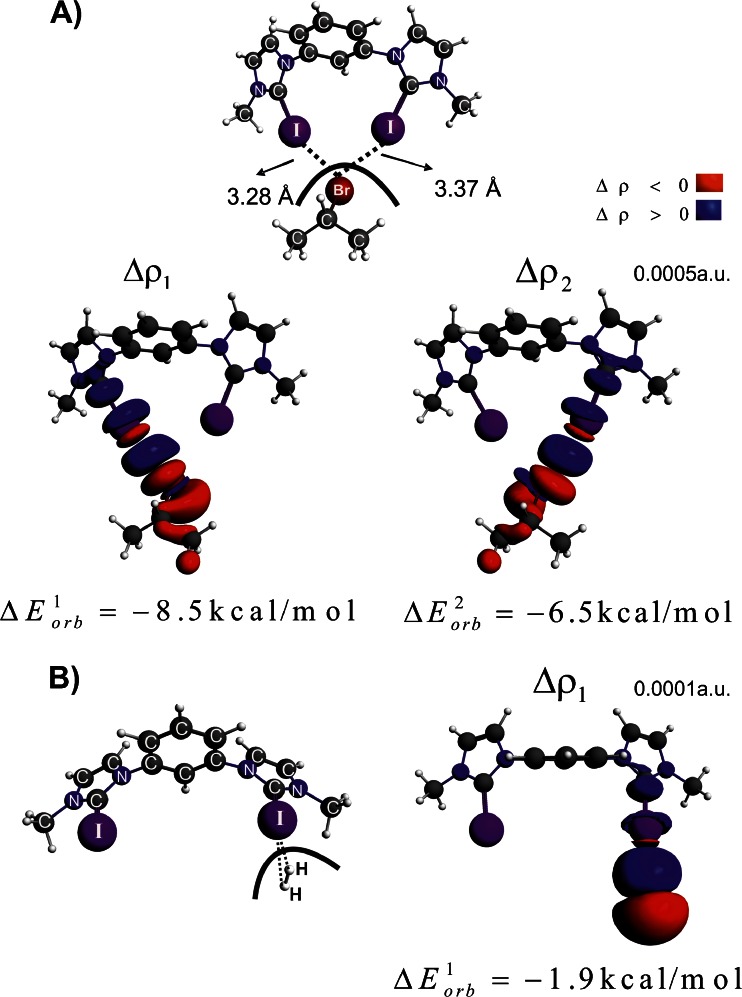
The contours of deformation density contributions describing the halogen bonding in **2-Br** (part A) and in **2-H**_**2**_ (part B). In addition the corresponding ETS-NOCV-based energies (in kcal mol^-1^) are shown

After we discussed the character of halogen bonding in simple molecules, let us consider now the bonding of isopropyl bromide to the model system of catalyst [C_6_H_4_(C_3_H_2_N_2_I)_2_][OTf]_2_, see **2-Br** in Fig. 1c. It has been proven experimentally that [C_6_H_4_(C_3_H_2_N_2_I)_2_][OTf]_2_ is able to break a strong carbon-bromide bond [39]. Authors of this work suggested that the activation of C–Br bond is induced by the formation of halogen bond between bromine and the iodine atoms, see Fig. 1c. We have found indeed, based on DFT/BP86-D/TZ2P calculations, the minimum on the potential energy surface for the complex **2-Br**, where the bromine center forms two halogen connections with iodine atoms. Energy decomposition method (ETS) indicates that isopropyl bromide is strongly bonded to [C_6_H_4_(C_3_H_2_N_2_I)_2_]^2+^ moiety, i.e., the total interaction energy is Δ*E*_int_ = -19.9 kcal mol^-1^. We found that C–Br bond becomes significantly elongated, by 0.08 Å (as compared to non-bonded isopropyl bromide), due to the formation of two Br–I bonds. Interestingly, in this case the orbital-interaction contribution (Δ*E*_orb_ = -19.6 kcal mol^-1^) appeared to be of the same importance compared to the electrostatic stabilization (Δ*E*_elstat_ = -20.3 kcal mol^-1^). It is an important result in light of the common view that the charge transfer (electronic factor) is a rather inferior factor in the halogen bonding [9, 21]. It is evident from Fig. 4a that two deformation density channels, Δ*ρ*_1_ and Δ*ρ*_2_, build up the two I–Br connections. ETS-NOCV allows to conclude that the one contribution is stronger ($\Delta E_{{orb}}^1 = {{{ - 8.5kcal}} \left/ {{mol}} \right.}$) than the other one ($\Delta E_{{orb}}^2 = {{{ - 6.5kcal}} \left/ {{mol}} \right.}$). It is consistent with the non-equivalent I–Br bond lengths, 3.28 Å and 3.37 Å, respectively. Figure 4a shows that, similar to the previously considered examples, both halogen bonds contain the covalent I–Br components as well as the charge transfer contributions originating from the lone electron pair donation to the empty σ*(C–I). Further mechanistic and kinetic studies leading to the heterolytic C–Br bond cleavage are under way.

Keeping in mind the above important experimental finding that a strongly electrophilic system can activate covalent bond, we decided to check whether the same system can form a chemical bond with chemically inert hydrogen molecule, H_2_. To our partial surprise we found a stable complex of dihydrogen with [C_6_H_4_(C_3_H_2_N_2_I)_2_]^2+^ moiety, see **2-H**_**2**_ in Fig. 1. ETS energy decomposition method indicated that the hydrogen unit is weakly bonded to iodine atom, Δ*E*_int_ = -1.9 kcal mol^-1^, see Table 1. Interestingly, neither the electrostatic term (Δ*E*_elstat_ = -1.6 kcal mol^-1^), nor the dispersion interaction (Δ*E*_disp_ = -1.0 kcal mol^-1^), are the leading contributions to Δ*E*_int_. It appears that the electronic factor (Δ*E*_orb_ = -2.2 kcal mol^-1^) is the most important for the bonding in **2-H**_**2**_, see Table 1. Deformation density channel originating from NOCV’s, Δ*ρ*_1_, presented in the right side of Fig. 4b, shows that the electronic stabilization originates predominantly from the electron donation from the occupied σ(H-H) orbital to the I–H bonding regions. This is further enhanced by σ-donation to the empty σ*(C–I) orbitals. We believe that this result is promising in terms of searching for new reactivity patterns of molecular hydrogen. It should be added that it has already been found that transition metal-based dihydride systems can form stable complexes with halogen-containing molecules [11].

**Fig. 5:**
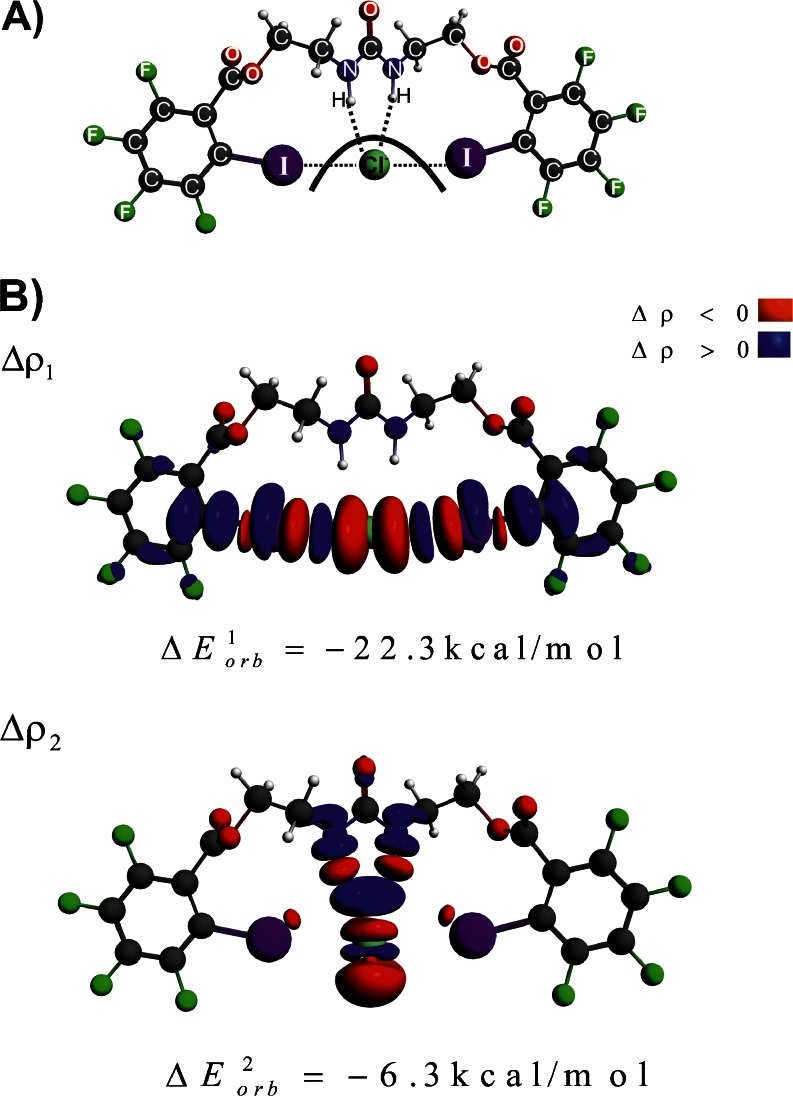
The contours of deformation density contributions describing the halogen (Δ*ρ*_1_) and hydrogen bonding (Δ*ρ*_1_) in **3-Cl**. In addition the corresponding ETS-NOCV-based energies (in kcal mol^-1^) are shown. The numerically smallest contour values are ±0.0005 a.u.

Finally, we will end our study by a brief discussion of the binding of chloride anion to a recently developed system that contains two iodine atoms and two N–H groups as electron acceptors, see **3-Cl** in Fig. 1. In line with expectation, the simultaneous presence of two hydrogen (Cl–HN) and two halogen (Cl–I) interactions, leads to very high total stabilization energy, Δ*E*_int_ = -62.4 kcal mol^-1^, see Table 1. It is the result of significant electrostatic (Δ*E*_elstat_ = -63.8 kcal mol^-1^) and electronic (Δ*E*_orb_ = -44.1 kcal mol^-1^) stabilizations. It is important to highlight that NOCV deformation density contributions combined with ETS scheme allowed to estimate separately the electronic strength of hydrogen (Cl–HN), Δρ_2_, and halogen (Cl–I) interactions, Δρ_1_, see Fig. 5b. It has been found that the halogen Cl–I bonding, in terms of the orbital interaction component, is more than three times stronger ($\Delta E_{{orb}}^1 = {{{ - 22.3kcal}} \left/ {{mol}} \right.}$) than the hydrogen bond connection ($\Delta E_{{orb}}^1 = {{{ - 6.3kcal}} \left/ {{mol}} \right.}$). Such separated information on the strength of halogen and hydrogen connections within the same molecule can help to modulate the selectivity and accordingly lead to rational design of new acceptor molecules.

## Conclusions

In the present study we have characterized the halogen bonding in selected molecules, **1-NH**_**3**_, **1-CPH**_**3**_, **2-Br**, **2-H**_**2**_ and **3-Cl**, based on the recently proposed ETS-NOCV procedure. We have chosen the examples containing from one (**1-NH**_**3**_, **1-CPH**_**3**_) up to four bonding connections (**3-Cl)**.

In the ETS energy decomposition scheme, the interaction energy between the fragments is divided into three well defined components: electrostatic, Pauli repulsion, and the orbital interaction. However, it should be emphasized that any energy partitioning method includes some arbitrariness due to the fact that the contributions to the total interaction energy are not physical observables. Nevertheless, the results of the present analysis, in agreement with previous studies [6–9, 57, 58] demonstrate the indisputable role of the electrostatic stabilization in halogen bonding.

We have found based on the NOCV-deformation density contours (Δ*ρ*_i_) that in each analyzed system the halogen bonding C–X^**…**^B (X-halogen atom, B-Lewis base), contains a large degree of covalent contribution (charge transfer to X–B inter-atomic region) supported further by the electron donation from base atom B to empty σ*(C-X) orbital. It should be emphasized that ETS-NOCV approach allowed to clearly visualize the formation of σ-hole at iodine atom of CF_3_I molecule. Thus, the NOCV-analysis confirms the vital importance of the presence of σ-hole for halogen bonding [6–9]; an electron-density deficiency at outer part of halogen atom (X) along C–X bond leads to both effects, electrostatic stabilization of the C–X^**…**^B interaction, but as well to a charge flow from a base to electron-deficient region.

An importance of the observed electron-density displacements is further demonstrated by the ETS orbital-interaction energy, Δ*E*_orb_, dominated in each case by one contribution Δ*E* ^*1*^_orb_ corresponding to main charge transfer channel Δ*ρ*_1_. Finally, in all of the analyzed systems the dispersion interaction appeared to be less important for halogen bonding.

Due to the fact that in each case we noted a large degree of covalency of halogen bond, we believe that the term “non-covalent interaction”, frequently used in literature, is not fully appropriate, at least for the systems studied in the present work. These results suggest further that in addition to the dominant electrostatic component, depending on the system, other terms can be relatively important. This observation supports the new set of criteria proposed recently by Legon for definition of halogen bonding [14].

We have also demonstrated that strongly electrophilic species, [C_6_H_4_(C_3_H_2_N_2_I)_2_][OTf]_2_, (**2**), can activate chemically inert molecules via formation of halogen bonding, e.g., isopropyl bromide (Δ*E*_int_ = -19.9 kcal mol^-1^). Interestingly, it has also been noted that (**2**) can bind the hydrogen molecule, **2-H**_**2**_, Δ*E*_int_ = -1.8 kcal mol^-1^, which seems to be a promising result in terms of future findings of new reactivity patterns of hydrogen molecule.

Finally, ETS-NOCV approach allowed to qualitatively and quantitatively characterize separately the halogen (Cl–I) and hydrogen bonds (Cl–HN) within the same acceptor molecule (**3-Cl**).
